# The Association Between Cancer and Dementia: A National Cohort Study in Sweden

**DOI:** 10.3389/fonc.2020.00073

**Published:** 2020-02-04

**Authors:** Ming Sun, Youxin Wang, Jan Sundquist, Kristina Sundquist, Jianguang Ji

**Affiliations:** ^1^Beijing Key Laboratory of Clinical Epidemiology, School of Public Health, Capital Medical University, Beijing, China; ^2^Center for Primary Health Care Research, Lund University/Region Skåne, Lund, Sweden; ^3^Department of Family Medicine and Community Health, Department of Population Health Science and Policy, Icahn School of Medicine at Mount Sinai, New York, NY, United States; ^4^Department of Functional Pathology, Center for Community-based Healthcare Research and Education (CoHRE), School of Medicine, Shimane University, Matsue, Japan

**Keywords:** cancer, dementia, inverse association, incidence, nationwide

## Abstract

**Background:** Previous studies have found that the incidence of dementia is lower in patients with cancer. However, the impact of survival bias, as well as the confounding by medical treatment, have not been fully addressed. We aimed to explore the subsequent risk of dementia in different follow-up intervals among patients with cancer, as well as the risk before the diagnosis of cancer.

**Methods:** By using the Swedish Cancer Register and the Swedish Hospital Discharge Register, we systematically examined the risk of dementia among patients diagnosed with 35 different types of cancer. Standardized incidence ratios (SIRs) were used to calculate the relative risk.

**Results:** The subsequent risk of dementia in patients with cancer decreased by 21% compared to matched cancer-free controls (SIR = 0.79, 95% CI 0.78–0.80). For specific cancer sites, 21 of them had a significantly lower risk of subsequent dementia. The decreased risk of dementia was also significant before the diagnosis of cancer. However, the risk was higher among patients with cancer who survived for more than 10 years' post-diagnosis (SIR = 1.37, 95% CI 1.32–1.41).

**Conclusions:** In this population-based study, we found that the risk of dementia was lower among patients with cancer, and the risk was also lower before the diagnosis of cancer. This suggests that lower dementia risk is not simply due to bias. However, the underlying mechanisms need to be explored further.

## Introduction

Several studies have demonstrated that there is an inverse association between cancer and dementia (1–7). An inverse association with cancer has also been found in other neurodegenerative diseases, such as Parkinson's disease (8–10). However, the underlying mechanisms are largely unknown. Previous studies suggested that dementia and many site-specific cancers share one or more common molecular mechanisms, such as signaling pathways and Pin enzyme (11, 12). Besides this, there are many other factors that might explain a link between cancer and dementia, including shared risk factors, age-related changes, and the possible effects of cancer treatment on the brain (13). Survival bias, which means that cancer survivors may be more likely to die before they can develop dementia, has been suggested to confound the observed association (2, 14). A potential mechanism is that biological pathway to cancer might be protective against dementia (15–18), but the existing evidence remains inconclusive. The observed inverse association is further complicated by the medical treatments of various types of cancer, which have been reported to be associated with either a higher or lower risk of dementia (5, 19–21).

In this population-based national study, we aimed to systematically explore the subsequent risk of dementia after the diagnosis of a total of 35 different cancers. The relative risk of dementia was stratified by follow-up time with a consideration of survival bias. We also explored the risk of dementia before the diagnosis of various cancers to control for the contribution by medical treatments of cancer. We further investigated the risk of dementia stratified by subtypes, i.e., Alzheimer's dementia (AD) or vascular dementia (VaD), which have a different underlying pathogenesis. AD is caused by neurodegenerative process whereas VaD is caused by cerebrovascular insults. This study, to the best of our knowledge, is the first nationwide large-scale study with more than 700,000 cancer cases included.

## Methods

This retrospective cohort study was approved by the Ethics Committee at Lund University, Sweden. This study was carried out by combining data from the Swedish Cancer Register and the Swedish Hospital Discharge Register. The Swedish Cancer Register was founded in 1958 and has almost complete nationwide coverage (22). We identified all malignancies according to the 7th Revision of International Classification of Diseases (ICD-7 codes 140–209), as used in the Swedish Cancer Register (Supplementary Table 1). The Swedish Hospital Discharge Register, which was founded in 1964, and has had complete nationwide coverage since 1987. All dementia patients were identified from the Swedish Hospital Discharge Register. In Sweden, dementia disorders are clinically diagnosed according to different versions of International Classification of Diseases, as used in the Swedish Hospital Discharge Register. Cognitive evaluation was performed using the mini-mental state examination (MMSE) scores (23). ICD-9 code of 290 was used to retrieve patients diagnosed with dementia in the years between 1987 and 1996. ICD-10 codes of F00-F03 and G30 were used for patients diagnosed between 1997 and 2015.

### Study Population

The study population includes people who were born before 1947 and were still alive in 1992 (older than 45 at the beginning of the study). Using the Swedish Cancer Register, all people were identified who were diagnosed with cancer from January 1, 1992, to December 31, 2015. Only the first primary cancer was considered in the present study. At least three cancer-free individuals were matched with patients with cancer according to year and month of birth, gender, highest education level, country of origin, history of diabetes and hypertension. Individuals who had a diagnosis of any dementia before 1987 were excluded.

### Covariates

By linking to the Total Population Register and the Hospital Discharge Register, we could identify a range of demographic and clinical factors that might confound our results. The covariates included in the study were gender (male/female), age at diagnosis and time period at diagnosis of dementia, which were categorized into 5-year groups, highest education level, country of origin, diabetes (yes/no), and hypertension (yes/no). The highest education level was classified into four categories: (1) <9 years, (2) 10–11 years, (3) >12 years, and (4) unknown. Country of origin was grouped as being born in Sweden and born abroad (24).

### Statistical Analysis

Person-year at risk was calculated from the year and month of cancer diagnosis until the diagnosis of dementia, death, or the end of follow-up (December 31, 2015), whichever came first. Standardized incidence ratios (SIRs) were calculated as the ratio of observed to expected number of cases. SIRs were used to measure the relative risk of dementia in individuals diagnosed with cancer compared with the matched cancer-free group. The expected number of cases was calculated based on age-, sex-, education-, country of origin-, period-, diabetes-, and hypertension-specific standard incidence rates derived from the matched cancer-free group. We excluded cancer types with <10 dementia cases. We calculated 95% CIs assuming a Poisson distribution.

To determine if there were substantial differences in the association between cancer and dementia by type of cancer or dementia subtypes, we stratified the main analysis according to anatomical site (oral, salivary gland, esophageal, stomach, small intestine, colon, rectum, anus, liver, pancreas, nose, lung, breast, cervix, endometrium, uterus, ovary, other female genital, prostate, testis, other male genital, kidney, urinary bladder, melanoma, skin, eye, nervous system, thyroid gland, endocrine glands, bone, connective tissue, non-Hodgkin's lymphoma, Hodgkin's lymphoma, myeloma, and leukemia) and dementia classification (AD, VaD, and other dementia). Cancer cases were also classified into smoking-related cancers (oral, esophageal, stomach, pancreas, lung, cervix, kidney, and urinary bladder) and non-smoking-related cancers based on the evidence from IARC (25). To explore the possibility of survival bias, we calculated the risk of dementia stratified by follow-up interval (0–4, 5–9, and ≥10 years; Supplementary Figure 1). We investigated the incidence of dementia 5 years before the diagnosis of cancer to control for the confounding effects, such as negative surveillance bias and medication. We also assess the robustness of our research findings to unmeasured confounding by using the e-value as suggested by VanderWeele and Ding (26). All analyses were performed using SAS version 9.3 (SAS Institute, Cary, NC, USA).

## Results

The demographic and clinical factors among patients with cancer and the matched controls are presented in Table 1. A total of 732,901 individuals diagnosed with cancer were retrieved from the databases. Male patients (55.3%) outnumbered female patients (44.7%). The median age at diagnosis of cancer was 73 years. After a mean of 5.46 years of follow-up, 14,090 (5.6‰) of the patients were diagnosed with dementia.

**Table 1:** Basic demographic and clinical characteristics among patients with cancer and cancer-free controls.

**Characteristics**	**Cancer group (*n* = 732901)**	**Cancer-free group (*n* = 1769357)**
**Gender**		
Male	405130 (55.3%)	863382 (48.8%)
Female	327771 (44.7%)	905975 (51.2%)
**Birth year**		
<1920	127770 (17.4%)	352275 (19.9%)
1920–1929	213316 (29.1%)	450453 (25.5%)
1930–1939	223622 (30.5%)	496071 (28.0%)
1940+	168193 (22.9%)	470558 (26.6%)
Median	1931	1931
**Birth country**		
Sweden	663124 (90.5%)	1571966 (88.8%)
Others	69777 (9.5%)	197391 (11.2%)
**Education (years)**		
1–9	341364 (46.6%)	815617 (46.1%)
10–11	227412 (31.0%)	530199 (30.0%)
12+	126581 (17.6%)	294792 (16.7%)
Unknown	37544 (5.1%)	128749 (7.3%)
**Diabetes**	114047 (15.6%)	261130 (14.8%)
**Hypertension**	289501 (39.5%)	659407 (37.3%)

The overall risk of dementia was significantly lower among patients with cancer (SIR = 0.79, 95% CI 0.78–0.80) as compared to the controls after adjusting for a range of factors (Table 2). For specific cancer sites, the lowest risk of dementia was found after the diagnosis of pancreatic cancer (SIR = 0.30, 95% CI 0.18–0.47), followed by esophageal cancer (SIR = 0.34, 95% CI 0.19–0.56) and liver cancer (SIR = 0.39, 95% CI 0.27–0.54). A total of 21 cancer sites showed an inverse association with dementia. The inverse association was stronger for smoking-related cancers (SIR = 0.76, 95% CI 0.72–0.79) than for non-smoking-related cancers (SIR = 0.80, 95% CI 0.78–0.80). The e-values for the point estimate and upper confidence bound for dementia were shown in Supplementary Table 2.

**Table 2:** Risk of dementia among patients with cancer stratified by anatomical site.

**Cancer types**	**O**	SIR^*^(95% CI)	
Oral (upper aerodigestive tract)	228	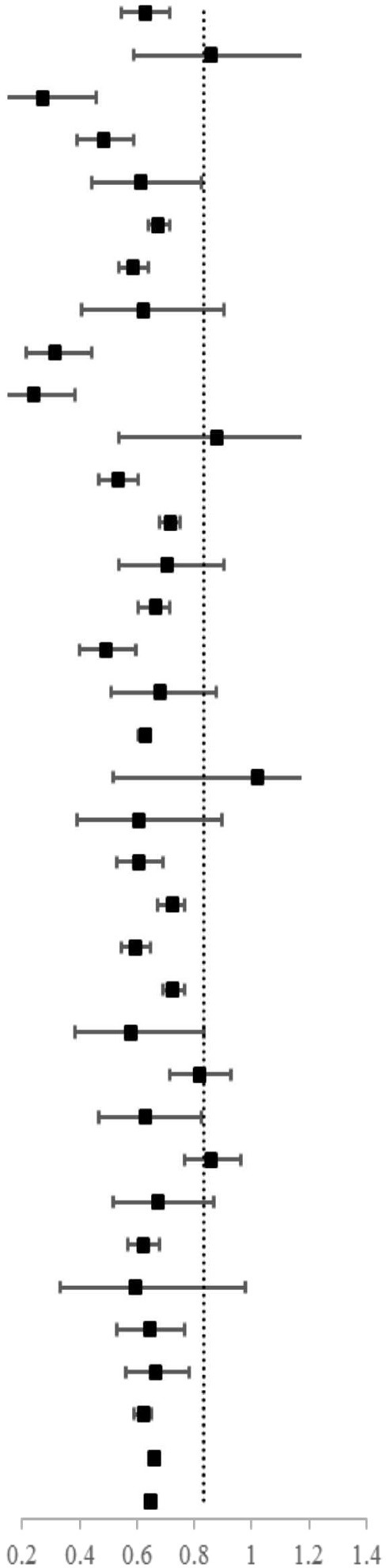	**0.76 (0.66–0.86)**
Salivary gland	34		1.03 (0.71–1.43)
Esophageal	16		**0.34 (0.19–0.56)**
Stomach	110		**0.59 (0.48–0.71)**
Small intestine	46		**0.74 (0.54–0.99)**
Colon	1,272		**0.81 (0.77–0.86)**
Rectum	568		**0.71 (0.65–0.77)**
Anus	29		0.75 (0.50–1.08)
Liver	34		**0.39 (0.27–0.54)**
Pancreas	18		**0.30 (0.18–0.47)**
Nose	21		1.05 (0.65–1.61)
Lung	260		**0.65 (0.57–0.73)**
Breast	2,124		**0.86 (0.82–0.90)**
Cervix	65		0.85 (0.65–1.08)
Endometrium	558		**0.80 (0.73–0.86)**
Ovary	113		**0.60 (0.49–0.72)**
Other female genitals	60		0.82 (0.62–1.05)
Prostate	3,724		**0.76 (0.73**–**0.78)**
Testis	12		1.22 (0.63–2.14)
Other male genitals	26		0.73 (0.48–1.07)
Kidney	251		**0.73 (0.64**–**0.83)**
Urinary bladder	972		**0.87 (0.81**–**0.92)**
Melanoma	538		**0.72 (0.66**–**0.78)**
Skin	1,478		**0.87 (0.83**–**0.92)**
Eye	30		0.70 (0.47–1.00)
Nervous system	228		0.98 (0.86–1.11)
Thyroid gland	54		**0.76 (0.57**–**0.99)**
Endocrine glands	319		1.03 (0.92–1.15)
Connective tissue	65		0.81 (0.63–1.04)
Non-Hodgkin's lymphoma	538		**0.75 (0.69**–**0.82)**
Hodgkin's disease	16		0.72 (0.41–1.17)
Myeloma	124		**0.78 (0.64**–**0.92)**
Leukemia	159		**0.80 (0.68**–**0.94)**
Smoking-related^#^	1920		**0.76 (0.72**–**0.79)**
Non-smoking-related	12,170		**0.80 (0.78**–**0.81)**
All	14,090		**0.79 (0.78**–**0.80)**

* *Adjusted for age, sex, education, birth country, period of dementia diagnosis, diabetes, and hypertension*.

# *Defined as cancer of the oral, esophageal, stomach, pancreas, lung, cervix, kidney, and urinary bladder*.

*Bold values indicate SIR less than 1 and 95% CI does include 1.00*.

The risk of dementia was further stratified by follow-up interval as shown in Table 3. Within 5 years before the diagnosis of cancer, the overall risk of dementia was 0.86 (95% CI 0.83–0.89). It was 0.60 (95% CI 0.58–0.61) during the first 5 years after the diagnosis of cancer, and 0.91 (95% CI 0.88–0.94) during the follow-up interval of 5–9 years. However, the overall risk of dementia was higher for cancer survivors who survived more than 10 years (SIR = 1.37, 95% CI 1.32–1.41). The lower risk was more evident in smoking-related cancer than in non-smoking-related cancer (SIR = 0.81 vs. 0.88, respectively) for patients who were diagnosed with dementia before the diagnosis of cancer. For specific cancer, we didn't find a lower risk of dementia during the whole follow-up period. For patients, who had survived for more than 5 years, only patients with rectal cancer (SIR = 0.85, 95% CI 0.69–0.97), breast cancer (SIR = 0.86, 95% CI 0.79–0.93), prostate cancer (SIR = 0.94, 95% CI 0.89–0.99), melanoma (SIR = 0.71, 95% CI 0.60–0.84), and non-Hodgkin's lymphoma (SIR = 0.82, 95% CI 0.69–0.97) showed a lower risk of dementia.

**Table 3:** Risk of dementia among patients with cancer stratified by different follow up period.

**Cancer types**	**Before the diagnosis**			**After the diagnosis**								
	**−5–0 years**			**0–4 years**			**5–9 years**			**≥10 years**		
	**O**	SIR^*^	**95% CI**	**O**	SIR^*^	**95% CI**	**O**	SIR^*^	**95% CI**	**O**	SIR^*^	**95% CI**
Oral (upper aerodigestive tract)	87	1.04	0.84–1.29	100	**0.55**	0.45–0.67	70	0.92	0.72–1.16	58	1.29	0.98–1.67
Salivary gland	7	0.78	0.31–1.62	13	0.69	0.37–1.19	11	1.26	0.62–2.26	10	1.77	0.84–3.24
Esophageal	56	1.17	0.88–1.52	12	**0.33**	0.17–0.57	2	0.31	0.03–1.15	2	0.55	0.05–2.04
Stomach	118	0.87	0.72–1.04	61	**0.47**	0.36–0.61	27	0.74	0.49–1.07	22	1.05	0.66–1.59
Small intestine	13	0.58	0.31–1.00	20	**0.51**	0.31–0.80	11	0.73	0.36–1.31	15	1.91	1.07–3.16
Colon	407	**0.81**	0.73–0.89	568	**0.60**	0.55–0.65	394	1.00	0.90–1.10	310	1.43	1.27–1.59
Rectum	188	**0.83**	0.70–0.96	259	**0.54**	0.48–0.61	170	**0.85**	0.72–0.98	139	1.17	0.98–1.38
Anus	19	1.56	0.94–2.44	11	**0.48**	0.24–0.85	10	1.04	0.50–1.93	8	1.33	1.57–2.64
Liver	102	**0.81**	0.66–0.99	17	**0.24**	0.14–0.38	10	0.95	0.45–1.75	7	1.15	0.46–2.38
Pancreas	107	0.87	0.71–1.05	16	**0.29**	0.17–0.48	2	0.57	0.05–2.09	0	-	-
Nose	7	1.08	0.43–2.24	8	0.64	0.27–1.26	6	1.15	0.41–2.51	7	3.20	1.27–6.62
Lung	239	**0.69**	0.61–0.78	154	**0.48**	0.41–0.57	64	1.12	0.86–1.43	42	1.56	1.12–2.11
Breast	598	1.17	1.08–1.27	788	**0.63**	0.59–0.68	580	**0.86**	0.79–0.93	756	1.39	1.29–1.49
Cervix	35	1.35	0.94–1.88	24	**0.59**	0.38–0.88	19	1.02	0.61–1.60	22	1.27	0.79–1.92
Endometrium	151	1.15	0.97–1.35	180	**0.54**	0.46–0.62	173	0.87	0.75–1.01	205	1.22	1.06–1.40
Ovary	44	**0.72**	0.52–0.97	33	**0.30**	0.20–0.42	36	0.87	0.61–1.21	44	1.21	0.88–1.63
Other female genitals	30	0.87	0.58–1.24	34	0.75	0.52–1.05	14	0.80	0.43–1.34	12	1.14	0.58–1.99
Prostate	569	**0.62**	0.57–0.67	1669	**0.55**	0.53–0.58	1293	**0.94**	0.89–0.99	762	1.42	1.32–1.53
Testis	3	2.86	0.54–8.48	3	0.75	0.14–2.23	5	2.00	0.63–4.71	4	1.20	0.31–3.09
Other male genitals	14	1.56	0.85–2.63	18	0.85	0.50–1.34	4	0.42	0.11–1.08	4	0.85	0.22–2.20
Kidney	44	**0.47**	0.34–0.63	105	**0.53**	0.43–0.64	80	0.90	0.72–1.12	66	1.20	0.93–1.52
Urinary bladder	251	**0.83**	0.73–0.93	436	**0.66**	0.60–0.72	286	0.98	0.87–1.10	250	1.50	1.32–1.70
Melanoma	197	1.16	1.00–1.33	223	**0.53**	0.46–0.61	141	**0.71**	0.60–0.84	174	1.28	1.10–1.49
Skin	627	1.07	0.99–1.16	839	**0.77**	0.72–0.83	388	0.92	0.83–1.02	251	1.33	1.17–1.50
Eye	6	0.68	0.24–1.48	7	**0.28**	0.11–0.58	9	0.80	0.36–1.53	14	2.07	1.13–3.48
Nervous system	77	1.27	1.00–1.59	89	**0.79**	0.64–0.98	61	0.95	0.73–1.22	78	1.38	1.09–1.72
Thyroid gland	9	**0.48**	0.22–0.91	14	**0.39**	0.21–0.65	21	1.11	0.68–1.70	19	1.18	0.71–1.84
Endocrine glands	50	1.29	0.95–1.70	129	0.93	0.78–1.10	93	1.03	0.83–1.27	97	1.20	0.97–1.46
Connective tissue	25	0.96	0.62–1.42	31	**0.65**	0.44–0.93	20	1.00	0.61–1.55	14	1.13	0.62–1.90
Non-Hodgkin's lymphoma	155	**0.67**	0.57–0.79	260	**0.57**	0.51–0.65	145	**0.82**	0.69–0.97	133	1.59	1.33–1.88
Hodgkin's disease	3	0.44	0.08–1.31	5	**0.40**	0.13–0.94	6	1.05	0.38–2.30	5	1.23	0.39–2.90
Myeloma	49	**0.66**	0.49–0.87	92	**0.71**	0.57–0.87	16	0.69	0.40–1.13	16	2.34	1.33–3.81
Leukemia	60	**0.58**	0.44–0.75	95	**0.69**	0.56–0.85	40	0.93	0.66–1.27	24	1.34	0.86–1.99
Smoking related^#^	937	**0.81**	0.76–0.86	908	**0.56**	0.52–0.60	550	0.95	0.87–1.03	462	1.37	1.25–1.50
Non-smoking related	3410	**0.88**	0.85–0.90	5405	**0.60**	0.59–0.62	3657	**0.91**	0.88–0.94	3108	1.37	1.31–1.41
All	4347	**0.86**	0.83–0.89	6313	**0.60**	0.58–0.61	4207	**0.91**	0.88–0.94	3570	1.37	1.32–1.41

* *Adjusted for age, sex, education, birth country, period of dementia diagnosis, diabetes, and hypertension*.

# *Defined as cancer of the oral, esophageal, stomach, pancreas, lung, cervix, kidney, and urinary bladder*.

*Bold values indicate SIR less than 1 and 95% CI does include 1.00*.

The risks of dementia by subtypes are presented in Table 4. The overall risk of AD was 0.82 (95% CI 0.80–0.85), 0.78 (95% CI 0.75–0.80) for VaD, and 0.78 (95% CI 0.76–0.80) for other dementia types. The lower risk was more evident in smoking-related cancer than in non-smoking-related cancer (SIR = 0.77 vs. 0.84, respectively) for patients who were diagnosed with AD. For specific cancer site, a total of 12 cancer sites showed an inverse association with all the three dementia subtypes.

**Table 4:** Risk of dementia among patients with cancer stratified by different kinds of dementia.

**Cancer types**	**Alzheimer's**			**Vascular dementia**			**Other dementia**		
	**O**	SIR^*^	**95% CI**	**O**	SIR^*^	**95% CI**	**O**	SIR^*^	**95% CI**
Oral (upper aerodigestive tract)	78	0.87	0.69–1.08	65	**0.70**	0.54–0.89	85	**0.73**	0.58–0.90
Salivary gland	18	1.76	1.04–2.79	5	0.50	0.16–1.18	11	0.85	0.42–1.52
Esophageal	3	**0.22**	0.04–0.64	4	**0.28**	0.07–0.71	9	**0.49**	0.22–0.93
Stomach	31	**0.58**	0.40–0.93	34	**0.59**	0.41–0.82	45	**0.61**	0.44–0.81
Small intestine	11	0.56	0.28–1.01	13	0.69	0.37–1.18	22	0.94	0.59–1.42
Colon	425	**0.90**	0.81–0.98	348	**0.74**	0.67–0.83	499	**0.80**	0.73–0.87
Rectum	185	**0.78**	0.67–0.90	160	0.65	0.56–0.76	223	**0.71**	0.62–0.81
Anus	8	0.63	0.27–1.24	7	0.66	0.29–1.37	14	0.98	0.54–1.61
Liver	9	**0.33**	0.15–0.63	9	**0.34**	0.15–0.64	16	**0.47**	0.27–0.77
Pancreas	1	0.05	0.00–0.30	4	**0.22**	0.06–0.58	13	**0.57**	0.30–0.98
Nose	5	0.82	0.26–1.92	10	1.70	0.81–3.14	6	0.75	0.27–1.64
Lung	90	**0.69**	0.56–0.85	65	**0.55**	0.42–0.70	105	**0.68**	0.56–0.83
Breast	824	**0.91**	0.85–0.97	527	**0.86**	0.79–0.94	773	**0.82**	0.76–0.88
Cervix	20	0.74	0.46–1.15	13	0.70	0.35–1.15	32	1.05	0.72–1.49
Endometrium	205	**0.83**	0.72–0.95	144	**0.78**	0.66–0.92	209	**0.78**	0.68–0.90
Ovary	54	0.80	0.60–1.04	22	**0.46**	0.29–0.70	37	**0.50**	0.35–0.69
Other female genitals	19	0.82	0.49–1.28	14	0.73	0.40–1.22	27	0.87	0.57–1.27
Prostate	1127	**0.80**	0.75–0.85	1262	**0.77**	0.73–0.82	1335	**0.72**	0.68–0.76
Testis	2	0.66	0.06–2.44	4	1.21	0.31–3.13	6	1.71	0.62–3.75
Other male genitals	10	1.03	0.49–1.91	7	0.58	0.23–1.21	9	0.65	0.29–1.24
Kidney	83	**0.78**	0.62–0.97	82	**0.76**	0.60–0.94	86	**0.69**	0.55–0.84
Urinary bladder	282	**0.87**	0.77–0.98	325	0.89	0.80–1.00	365	**0.85**	0.76–0.94
Melanoma	175	**0.70**	0.60–0.81	158	**0.72**	0.61–0.84	205	**0.74**	0.64–0.85
Skin	448	**0.89**	0.81–0.98	448	**0.90**	0.82–0.98	582	**0.84**	0.78–0.92
Eye	11	0.80	0.40–1.43	7	0.55	0.22–1.14	12	0.72	0.37–1.27
Nervous system	59	**0.73**	0.55–0.94	51	0.77	0.57–1.02	118	1.39	1.15–1.66
Thyroid gland	16	0.66	0.38–1.07	16	0.79	0.45–1.29	22	0.82	0.51–1.24
Endocrine glands	102	1.00	0.82–1.22	94	1.03	0.83–1.26	123	1.07	0.89–1.27
Connective tissue	16	0.66	0.38–1.07	22	0.92	0.57–1.39	27	0.85	0.56–1.24
Non-Hodgkin's lymphoma	164	**0.73**	0.62–0.85	147	**0.70**	0.59–0.83	227	**0.82**	0.72–0.93
Hodgkin's disease	5	0.69	0.22–1.63	6	0.88	0.32–1.93	5	0.61	0.19–1.42
Myeloma	37	0.76	0.54–1.05	34	**0.71**	0.49–0.99	53	0.84	0.63–1.09
Leukemia	52	0.82	0.61–1.08	50	0.87	0.64–1.15	57	**0.76**	0.58–0.99
Smoking related^#^	588	**0.77**	0.71–0.84	592	**0.75**	0.69–0.81	740	**0.76**	0.71–0.82
Non-smoking related	3987	**0.84**	0.81–0.86	3565	**0.78**	0.76–0.81	4618	**0.78**	0.76–0.81
All	4575	**0.82**	0.80–0.85	4157	**0.78**	0.75–0.80	5358	**0.78**	0.76–0.80

* *Adjusted for age, sex, education, birth country, period of dementia diagnosis, diabetes, and hypertension*.

# *Defined as cancer of the oral, esophageal, stomach, pancreas, lung, cervix, kidney, and urinary bladder*.

*Bold values indicate SIR less than 1 and 95% CI does include 1.00*.

## Discussion

In this population-based nationwide cohort study, we found that the incidence of dementia was significantly lower among patients with cancer as compared to the matched cancer-free group. The overall risk of dementia continued to be significantly lower until 10 years of follow-up, suggesting that the lower risk of dementia may only partially lie behind survival bias. Furthermore, the decrease in dementia risk was also significantly before the diagnosis of cancer, which suggests that the observed inverse association might represent a “true” finding, rather than simply an artifact of survival bias or under-diagnosis.

The inverse relation between cancer and dementia was found in a total of 21 cancer sites in our study. Our finding shows that the inverse relation was more evident in smoking-related cancers than non-smoking-related cancers for patients who were diagnosed with AD, which was consistent with previous report (2), but similar in other subtypes of dementia. Smoking is a well-known risk factor for both dementia and a few types of cancer (27); we thus can speculate that the observed inverse association between cancer and dementia cannot entirely be explained by smoking-related factors. Other vascular-related factors, such as hypertension and diabetes (28), were also included in the analytical model; thus, these factors may play a minor role in the observed association. In addition, the observed inverse associations were noted in all dementia subtypes, including AD, VaD, and other dementias; thus, the contribution by vascular-related factors can be further excluded. When we stratified the risk of dementia by follow-up interval, the inverse association was observed until 10 years of follow-up. In addition, the risk of dementia was also lower before the diagnosis of cancer, suggesting that survival bias and medical treatments of cancer cannot explain the observed negative association.

Our data suggested that the observed inverse association between dementia and cancer might be a true finding, but the underlying mechanisms are still largely unknown (15). Differential regulation of common genes and pathways might be related to the inverse association between cancer and dementia. Abnormal cell behaviors are the essence of both cancer and dementia (29). Cancer is characterized by unlimited cellular proliferation, whereas dementia is a process of premature cell death. Previous studies have shown that cancer and dementia may have shared genes and biological pathways, but these are often regulated in different directions (11, 30). A genome-wide association study found significant genetic associations between cancer and dementia, and some gene expression regulators have opposite regulatory effects on cancer and dementia (15). Previous studies have also found overlapping genes and signaling pathways between cancer and dementia (12). Pin1 enzyme plays a critical but opposite role in cancer and dementia (31–34). Over-expression of Pin1 can promote tumorigenesis as shown in cancers of the prostate, lung, breast, and so on (33). However, the overexpression of Pin1 gene in nerve cell has a neuroprotective effect by restoring phosphorylated tau and amyloid precursor protein to a functional state (31). Another gene that is regulated differentially in cancer and dementia is tumor suppressor protein P53, which is usually expressed less in patients with cancer but overexpressed in patients with dementia (35).

Medical treatment of cancer might confound the association between cancer and dementia. In our study, patients who survived more than 10 years had a higher risk of dementia as compared to the matched controls thus suggesting that medical treatment of cancer could influence the development of dementia. This may due to that chemotherapy can impair cognitive function, and patients with chemotherapy can have short-term or long-term changes in brain structure and function (13). Animal studies have indicated that some drugs might have neuroprotective effects (21, 36). A study on patients with breast cancer using the Surveillance, Epidemiology and End Results (SEER)-Medicare database showed no significant association between chemotherapy and cognitive impairment, but the incidence of AD and VaD was reduced (20). Another study that used the same database showed a possibility of severe cognitive changes associated with chemotherapy, particularly over a longer follow-up (13). Based on the evidence above, we can speculate that the effect of chemotherapy on the development of dementia might be complicated, which might depend on the doses and the types of different chemotherapeutical drugs. Unfortunately, information about medical treatment for cancer is not available in our databases, which calls for further studies using extended databases to explore their contributions. In addition, the explanation for the higher risk of dementia for cancer survivors after following up for more than 10 years might be due to aging-related cognitive decline, as well as continuous stress during the long-standing survival.

There are some strengths and limitations of this population-based study. A key strength of our study is that this is the first nationwide large-scale study to investigate the association between cancer and dementia by linking several nationwide Swedish registers. All the patients with cancer and patients with dementia were identified from the nationwide databases with high accuracy and high coverage (37). Using the data of nationwide registers can guarantee the completeness of the follow-up. A limitation of this study is that we lack information of other individual-related factors, such as polymorphisms association with dementia, smoking and physical activity. However, the e-value indicated that the observed SIR of 0.79 for overall dementia could only be explained by an unmeasured confounder that was associated with both cancer and dementia by a risk ratio of more than 1.85. Given that this risk ratio is greater than any observed for known dementia risk factors examined in the previous study, such as hypertension, diabetes, or smoking (38), it is implausible that an unmeasured confounder might explain the observed findings.

In conclusion, this population-based national cohort study suggested that the inverse association between cancer and dementia could not be due to survival bias or under-diagnosis. Further studies are needed to explore the underlying mechanisms.

## Data Availability Statement

The datasets generated for this study are available on request to the corresponding author.

## Ethics Statement

This retrospective cohort study was approved by the Ethics Committee at Lund University, Sweden.

## Author Contributions

MS, YW, JJ, KS, and JS were responsible for the study concept and design. JS, KS, JJ, and YW obtained funding. KS and JS acquired the data. MS did the statistical analysis and drafted the manuscript. All authors revised it for important intellectual content.

### Conflict of Interest

The authors declare that the research was conducted in the absence of any commercial or financial relationships that could be construed as a potential conflict of interest.
