# Supplementary material for: The Association Between Cancer and Dementia: A National Cohort Study in Sweden
Source: Front Oncol. 2020 Feb 4;10:73. doi: 10.3389/fonc.2020.00073 (PMC7010720; doi:10.3389/fonc.2020.00073)
Supplement: Supplementary file 1 [file Table_1.DOCX]

Supplementary Figure 1. Timeline of the study.

**
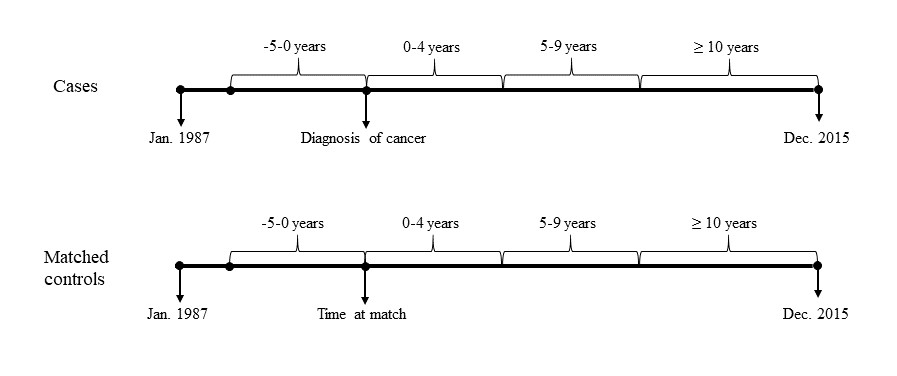
**

Supplementary Table 1. Definition and number of cancer types.

| Cancer types | ICD-7 codes | Number |
| --- | --- | --- |
| Oral (upper aerodigestive tract) | 140, 141, 143-148, 161 | 13874(1.9%) |
| Salivary gland | 142 | 1284(0.2%) |
| Oesophageal | 150 | 6796(0.9%) |
| Stomach | 151 | 17571(2.4%) |
| Small intestine | 152 | 3315(0.5%) |
| Colon | 153 | 60712(8.3%) |
| Rectum | 154 (except 1541) | 31868(4.4%) |
| Anus | 1541 | 1725(0.2%) |
| Liver | 155, 156 | 16697(2.3%) |
| Pancreas | 157 | 17248(2.4%) |
| Nose | 160 | 946(0.1%) |
| Lung | 162, 163 | 57763(7.9%) |
| Breast | 170 | 87300(11.9%) |
| Cervix | 171 | 4143(0.6%) |
| Endometrium | 172 | 22253(3.0%) |
| Uterus | 173 | 6(0.0%) |
| Ovary | 175 | 12022(1.6%) |
| Other female genital | 176 | 3405(0.5%) |
| Prostate | 177 | 152624(20.8%) |
| Testis | 178 | 446(0.1%) |
| Other male genital | 179 | 1314(0.2%) |
| Kidney | 180 | 16721(2.3%) |
| Urinary bladder | 181 | 39584(5.4%) |
| Melanoma | 190 | 25137(3.4%) |
| Skin | 191 | 49455(6.8%) |
| Eye | 192 | 1600(0.2%) |
| Nervous system | 193 | 14334(2.0%) |
| Thyroid gland | 194 | 3142(0.4%) |
| Endocrine glands | 195 | 9397(1.3%) |
| Bone | 196 | 538(0.1%) |
| Connective tissue | 197 | 3742(0.5%) |
| Non-Hodgkin's lymphoma | 200, 202 | 32053(4.4%) |
| Hodgkin's lymphoma | 201 | 1176(0.2%) |
| Myeloma | 203 | 10161(1.4%) |
| Leukaemia | 204, 209 | 12549(1.7%) |

Supplementary Table 2. E-value

| Cancer types | E-value for the point estimate | E-value for the upper CI |
| --- | --- | --- |
| Oral (upper aerodigestive tract) | 1.96 | 1.60 |
| Salivary gland | 1.21 | 1.00 |
| Oesophageal | 5.33 | 2.97 |
| Stomach | 2.78 | 2.17 |
| Small intestine | 2.04 | 1.11 |
| Colon | 1.77 | 1.60 |
| Rectum | 2.17 | 1.92 |
| Anus | 2.00 | 1.00 |
| Liver | 4.57 | 3.11 |
| Pancreas | 6.12 | 3.68 |
| Nose | 1.28 | 1.00 |
| Lung | 2.45 | 2.08 |
| Breast | 1.60 | 1.46 |
| Cervix | 1.63 | 1.00 |
| Endometrium | 1.81 | 1.60 |
| Ovary | 2.72 | 2.12 |
| Other female genitals | 1.74 | 1.00 |
| Prostate | 1.96 | 1.88 |
| Testis | 1.74 | 1.00 |
| Other male genitals | 2.08 | 1.00 |
| Kidney | 2.08 | 1.70 |
| Urinary bladder | 1.56 | 1.39 |
| Melanoma | 2.12 | 1.88 |
| Skin | 1.56 | 1.39 |
| Eye | 2.21 | 1.00 |
| Nervous system | 1.16 | 1.00 |
| Thyroid gland | 1.96 | 1.11 |
| Endocrine glands | 1.21 | 1.00 |
| Connective tissue | 1.77 | 1.00 |
| Non-Hodgkin's lymphoma | 2.00 | 1.74 |
| Hodgkin's disease | 2.12 | 1.00 |
| Myeloma | 1.88 | 1.39 |
| Leukaemia | 1.81 | 1.32 |
| Smoking-related^#^ | 1.96 | 1.85 |
| Non-smoking-related | 1.81 | 1.77 |
| All | 1.85 | 1.81 |
